# An Approach to the Fatigue in Young Soccer Players Resulting from Sided Games

**DOI:** 10.3390/sports7070174

**Published:** 2019-07-18

**Authors:** Daniel Castillo, Javier Yanci, Silvia Sánchez-Díaz, Javier Raya-González

**Affiliations:** 1 Faculty of Health Sciences, Universidad Isabel I and Burgos, 09003 Burgos, Spain; 2 Physical Education and Sport Department, Faculty of Education and Sport, University of Basque Country (UPV/EHU), 01007 Vitoria, Spain

**Keywords:** football, training context, quantification, perceived exertion, sprinting performance

## Abstract

It is crucial to understand the fatigue associated with sided games (SGs) of soccer in the training context, in order to establish the appropriate intervals between training sessions. Thus, the aim of this study was to investigate the effects of different SGs on internal load, measured by the session rating of perceived exertion (sRPE), and on sprint performance. Ten outfield players (age: 14.5 ± 0.5 years, height: 169 ± 6 cm, body mass: 59.7 ± 6.4 kg) belonging to U15 age category participated in this study. The participants played four SG formats with modifications in the pitch size and in the bout duration, but with the same total duration for the SGs (SG1, SG2, SG3, and SG4). All the players performed a 10 and a 30 m sprint test before and after the SGs. The internal load was measured by the sRPE. The results showed no significant differences (*p* > 0.05) in the sRPE registered by the soccer players for the different SGs, but worse sprint performances over the 10 m (*p* < 0.05; *ES*: 0.74–1.38, large) and 30 m (*p* < 0.05; *ES*: 0.70–2.10, moderate to large) distances after completion of the SGs, except the 10 m sprint after SG2 and SG3 (*p* > 0.05; *ES*: 0.43–0.55, moderate). In addition, no correlation (*p* > 0.05) was reported between the sprint performances for the 10 and 30 m distances and the sRPE registered during the SGs. These results could be useful for technical staff wishing to design the playing area and bout duration of their training tasks effectively.

## 1. Introduction

Sided games (SGs) are a useful strategy for technical staff in the training context, because these training games have a similar structure to the real game in terms of being a duel between teams, involving simultaneous activity and cooperation–opposition and taking place in an oriented space [1]. In spite of the fact that SGs are played in reduced playing areas, performing these tasks allows players to develop specific technical and tactical qualities and to improve their physical fitness [2,3]. Taking into consideration coaches’ needs and objectives, SGs can be played in different formats, through the modification of various parameters such as the individual interaction space (IIS), the playing time, the orientation of the playing area, the number of touches, the rules, the type of marking, the encouragement of the coaches, or the order in which the tasks are presented [4,5,6,7,8,9]. In fact, the modification of these parameters can influence the physical and physiological responses [10,11] and the perceived exertion of the soccer players [12]. This aspect is crucial in the design of SGs and for deciding on the intervals between training sessions within the microcycle.

Contradictory results have been obtained in the literature that has focused on the influence of different SG formats on the perceived exertion (PE) declared by soccer players [4,13,14]. Specifically, regarding the playing area, Casamichana and Castellano [4] demonstrated that, as the IIS was increased from small (75 m^2^) to medium (175 m^2^) to large (275 m^2^), the players declared higher PE values. In contrast, although other authors [14] have reported similar differences in physical response for SGs played in an IIS of 56 m^2^ and other SGs played in IISs of 87 m^2^ and 126 m^2^, they did not observe differences in the PE declared by the players for a medium SG and a large SG. It seems that there is no consensus in the literature about the nomenclature in terms of the dimensions of the game space in an SG, with it being necessary to use the term IIS to refer to the different SG formats. On the other hand, when considering the duration of the SGs, Fanchini et al. [13] did not observe any differences in the PE declared by players for a bout duration of four minutes and one of six minutes. While these investigations were carried out with senior soccer players, it would be interesting to deepen the analysis of the possible differences in the PE depending on the playing area, the bout duration and the combination of these two parameters in the design of the SG, because of the scarce knowledge about the influence of these different aspects of SGs on PE in lower age categories. Providing a deeper knowledge in relation to the fatigue resulting from SGs could be relevant for an understanding of the effects of SGs and for setting the intervals between the training sessions.

In addition to understanding the subjective responses to the different SGs, it could be interesting to know about the fatigue [15]. This knowledge would allow coaches to set the intervals between the training more appropriately during the microcycle. In order to determine the fatigue related to SGs, it is not enough to analyze the objective or subjective responses of the players during an SG. One must also determine their physical performance before and after the completion of the SG [7]. For example, one study [16] has shown that male college players had a lower sprint performance over distances of 5 m and 15 m (ES = 0.87 ± 0.58 and 0.89 ± 0.58, respectively) after a 4 vs. 4 format plus goalkeepers SG that lasted for three bouts of six minutes and was played on a pitch of size 48 × 30 m. In addition, Muñoz, Castillo, and Yanci [17] observed a loss of physical performance in the 5 m sprint after an SG played on a 31 × 19 m pitch that lasted for four bouts of three minutes with three minutes of rest, but no significant differences were found in sprint performance before and after the SG for other SG formats.

Maintaining sprinting performance throughout a match has been associated with optimizing on-field performance and reducing injury risk [18]. Therefore, the main aim of this study was to investigate the effects of different sided games (SGs) on the internal load, as measured by the session rating of perceived exertion (sRPE), and the loss of sprint performance. Taking into account the studies mentioned above, the hypothesis of this study is that a loss of sprint performance over 10 and 30 m distances will be found after the performance of different SGs.

## 2. Materials and Methods

### 2.1. Participants

Ten young male soccer players (age: 14.5 ± 0.5 years, height: 169 ± 6 cm, body mass: 59.7 ± 6.4 kg) in the Spanish under-15 age category took part in the study (statistical power > 0.80). The participants were involved in training three times per week, and played an official match every weekend. In addition, the players had been in the soccer club for 4.3 ± 3.1 years. The inclusion criteria were not having been injured during the month before the research and participating in the four SGs described in the research protocol. The coaches, players and parents or legal guardians were informed of the research procedures, requirements, benefits, and potential risks before providing their written informed consent (parents) and assent (players). The players participated voluntarily, and it was possible for them to withdraw from the investigation at any time without penalty. The study was performed in accordance with the Declaration of Helsinki (2013), and was approved by the Ethics Committee of the University of the Basque Country (UPV/EHU).

### 2.2. Study Design

**Figure 1:**
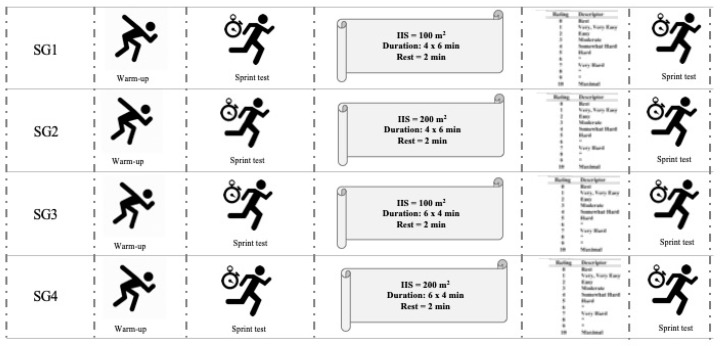
Temporal sequence of the experimental protocol during the investigation. **IIS:** individual interaction space; **SG1:** sided game played in an IIS of 100 m^2^ in 4 bouts of 6 min; **SG2:** sided game played in an IIS of 200 m^2^ in 4 bouts of 6 min; **SG3:** sided game played in an IIS of 100 m^2^ in 6 bouts of 4 min; and **SG4:** sided game played in an IIS of 200 m^2^ in 6 bouts of 4 min.

The study was carried out at the end of the 2017–2018 competitive season. The players took part in four different formats of SG at least 72 h after their previous official match. In addition, the players were instructed to sleep for at least eight hours the night before each data collection session, and to maintain adequate hydration and carbohydrate intake over the 24 h before each experimental SG. All the SGs took place at the same time (i.e., 8.30 p.m.) and on the same artificial grass pitch. Figure 1 shows the temporal sequence of the SGs that were held, and the sprint performance measures. At first, the players undertook a standardized warm-up, consisting of five minutes of slow jogging and strolling, followed by eight minutes of specific soccer drills, and finishing with three minutes of progressive sprints and accelerations. Second, the players performed a sprint test over a distance of 30 m. Thirdly, the participants took part in each format of SG with specific characteristics for IIS and bout duration. Fourthly, they declared their PE immediately after the end of each SG. Finally, the players again performed the sprint test over a distance of 30 m in a 40 s period. During the SG intervention, the players wore their normal soccer boots, and the researchers gave verbal encouragement.

#### 2.2.1. Sided Games (SGs)

Four different SG formats were proposed, with variations to the playing area and the duration of the bouts. The characteristics of the SGs are described in Figure 1 and were as follows: SG1, four bouts of six minutes in an IIS of 100 m^2^ (37 × 22 m); SG2, four bouts of six minutes in an IIS of 200 m^2^ (52 × 31 m); SG3, six bouts of four minutes in an IIS of 100 m^2^ (37 × 22 m); and SG4, six bouts of four minutes in an IIS of 200 m^2^ (52 × 31 m). These playing areas were chosen with the aim of conforming to the length–width ratio of a soccer pitch, which is 102 × 62 m. All the SGs had five outfield players and one goalkeeper per team, with the objective being to score more goals than the opponent. The offside rule applied. Because of their specific role on the field, the goalkeepers were not included in the analyses. The teams remained the same during the four sessions and used the same formation (i.e., one goalkeeper, one central defender, two wingers, one midfielder, and one forward) [6]. The coach was required to give verbal encouragement and to provide a new ball as soon as the ball went off the pitch, in order to increase the effective time [19].

#### 2.2.2. Sprint Performance

The participants performed three maximal repetitions of a sprint test over a distance of 30 m on an artificial grass field, interspersed with a 90 s rest period between sprints [7]. The players’ starting position was 0.5 m behind the first timing gate (Witty Microgate™, Microgate, Bolzano, Italy). The timer was automatically activated as the participant passed the first gate, that is, at the zero line. The time was recorded at 10 m (Sprint 10 m) and 30 m (Sprint 30 m) distances, and each player performed three trials with a 90 s rest period in between. The players wore their normal soccer boots. The best performance was then used for analysis. The Witty Manager software (Microgate, Bolzano, Italy) was used to download the sprint test data. The coefficient of variation (CV) for the 10 m and 30 m sprint was 2.26% and 0.78%, respectively, and the intra-class correlation coefficient (ICC) was 0.905 and 0.989 for the two physical tests.

#### 2.2.3. Perceived Exertion

In order to quantify the subjective responses of the soccer players during the SGs, the 10-point scale proposed by Foster et al. [20] was administered after the SGs had been played. The players answered the question: “*how hard was the SG*?” immediately after every SG [21]. The players were allowed to mark a plus sign (interpreted as half a point) alongside the integer value, and were not aware of their teammates’ PE scores. The same person (i.e., the researcher) was responsible for asking the players the question, without the other teammates being present. In addition, to calculate the session rating perceived exertion load (sRPEL), each score was multiplied by the duration of each SG, that is, 24 min for all the SG formats [22]. Moreover, (a) training monotony (the mean internal training load, measured as the sRPE across each SG divided by the standard deviation (SD)) and (b) training strain (the sum of the internal training loads measured by sRPE across each SG multiplied by the training monotony) were calculated [23]. The players became fully accustomed to the PE procedures during the competitive season.

### 2.3. Statistical Analysis

The results are presented as mean ± standard deviation (SD). Normality was tested using the Shapiro–Wilk test, and statistical parametric techniques were applied. The repeated-measures analysis of variance, with the Bonferroni post-hoc test, was used to compare the internal load measured by sRPEL between the SG formats. A *t*-test for paired samples was used to analyze the differences between the post-SG and pre-SG sprint performance measures. The percentage changes (Δ%) between the post-SG and pre-SG performances were calculated by the formula: Δ% = ([post-SG-pre-SG]/pre-SG) × 100. Statistical significance was set at *p* < 0.05. Practical differences were calculated using Cohen’s *d* effect size (ES, large: > 0.8; moderate: between 0.8 and 0.5; small: between 0.5 and 0.2; trivial < 0.2) [24]. In addition, Pearson’s product moment correlation coefficients (corresponding to 90% confidence intervals, CI) were calculated to determine whether there was a relationship between the internal load during the SG and the changes in sprint performance from pre-SG to post-SG. To interpret the results, the threshold values for the Pearson product–moment used by Salaj and Markovic [25] were used: Low (*r* ≤ 0.3), moderate (0.3 < *r* ≤ 0.7), and high (*r* > 0.7). The data analysis was carried out using the Statistical Package for Social Sciences (SPSS 25.0, SPSS™ Inc, Chicago, IL, USA).

## 3. Results

**Figure 2:**
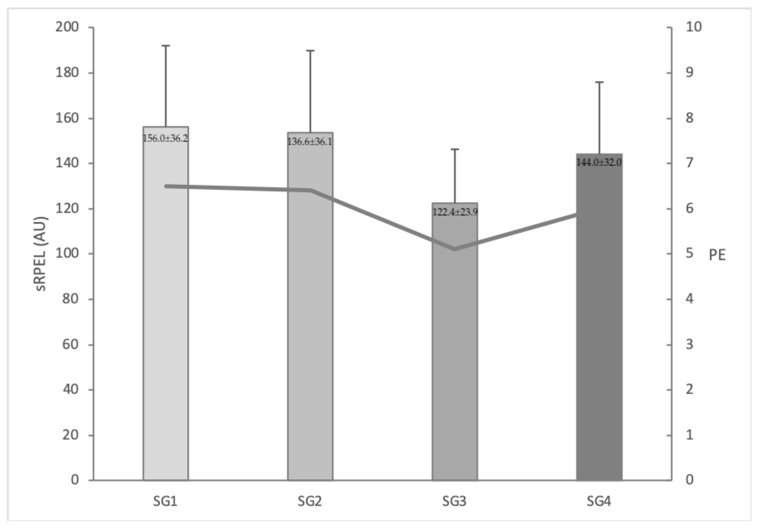
Session rating of perceived exertion load (sRPEL) (in columns) and perceived exertion (PE) (in line) registered for each sided game (SG). **SG1:** sided game played in an IIS of 100 m^2^ in 4 bouts of 6 min; **SG2:** sided game played in an IIS of 200 m^2^ in 4 bouts of 6 min; **SG3:** sided game played in an IIS of 100 m^2^ in 6 bouts of 4 min; and **SG4:** side game played in an IIS of 200 m^2^ in 6 bouts of 4 min.

Figure 2 shows the internal load measures by sRPEL and the PE values for each SG. No significant differences (*p* > 0.05) were found in the sRPEL (ES: 0.06–0.33, trivial to small) or PE (ES: 0.06–0.33, trivial to small) scores registered by the soccer players between the different SGs.

Table 1 presents the effects on sprint performance of each SG format. The soccer players increased the time it took to cover both 10 m (*p* < 0.05; *ES*: 0.74–1.38, large) and 30 m (*p* < 0.05; *ES*: 0.70–2.10, moderate to large) after all the SGs, except for the time to sprint 10 m after SG2 and SG3 (*p* > 0.05; *ES*: 0.43–0.55, moderate).

The training monotony was 4.31 AU for SG1, 4.25 AU for SG2, 5.13 AU for SG3 and 4.50 AU for SG4, and the strain was 671.86 AU for SG1, 652.95 AU for SG2, 627.74 AU for SG3 and 648.00 AU for SG4.

Finally, the associations between the percentage change (from pre- to post-SG) in the sprint performances over 10 and 30 m distances and the sRPEL registered during the SGs were not significant (*p* > 0.05) for any SG.

## 4. Discussion

The aim of this study was to analyze the influence of different SGs on the internal load, measured by the sRPEL, and on the variation in sprint performance. The main strength of this study was the combination of two different factors (i.e., duration and playing space) on the SGs, and the measurement of the possible influence of this on neuromuscular fatigue in young soccer players. The results showed no significant differences in the sRPEL registered by the soccer players among the different SGs, but worse sprint performances over a 30 m distance after the SGs had been played. In addition, no associations were reported between the delta changes from pre-SG to post-SG in sprint performances over 10 and 30 m distances or the sRPEL registered during the SGs. This study highlighted the necessity of analyzing fatigue for different SG formats.

Quantifying sRPEL during SGs is a cheap, useful and easy method for the technical staff of lower age categories of soccer teams [26]. Some authors have shown that players have declared subjective PE values of 6.7 ± 0.8, 6.7 ± 0.8 and 5.7 ± 1.0 in IISs of 75, 175 and 275 m^2^, respectively [4]. Other authors [14] observed similar values (6.2 ± 0.8 and 6.2 ± 0.6 in IISs of 87 and 126 m^2^, respectively). However, in our study, lower PE values (~5.5 AU) were recorded in all SG formats. These differences may be due to the inclusion of the offside rule in our investigation, in order to ensure the SGs resembled the real game, as this implies a better playing structure for the players on the soccer field [27,28]. Including the offside rule allows coaches to teach the principles of the game in the offensive and defensive phases with regards to the structural and functional parameters as well as the rules of soccer. For this reason, future investigations could study the effect of SGs with the offside rule in comparison to those without it on the physical, physiological and subjective demands on players.

Knowing the subjective PE of players during different training tasks is interesting for coaches because it allows them to set the intervals between sessions in the weekly training and to establish the training tasks in the microcycle in a more efficient way [29,30]. In regards to this aspect, no significant differences were reported in our study in the sRPEL registered by the soccer players between the different SG formats in which there were variations not only in the playing area (i.e., 100 or 200 m^2^), but also in the duration of the bouts (i.e., 4 or 6 min). Along the same lines, Fanchini et al. [13] observed that the PE was not affected by the duration of the bouts (i.e., 2, 4, or 6 min). However, Muñoz et al. [17] showed higher respiratory PE scores for an SG played in an IIS of 75 m^2^ than for the same SG format played in an IIS of 175 m^2^. By contrast, other studies have demonstrated that different SG formats can influence the physical responses of soccer players [6,8], although it seems that the players did not declare different subjective responses. Taking these results into account, coaches should discuss these parameters in order to establish, in a more appropriate way, the total duration of the SGs and the duration of each bout.

Quantifying the subjective demands registered by the players during the playing of SGs is not sufficient to assess their fatigue, as it is also necessary to analyze their performance in certain physical tests before and after the SGs [7]. Contradictory results have been reported in the scientific literature about how different SG formats affect performance in the sprint test. On the one hand, Rebelo et al. [16] showed that players took longer to cover distances of 5 m and 15 m after SGs played in a format of 5 vs. 5 (goalkeepers included) in an IIS of 140 m^2^, and Castillo et al. [7] found a worse sprint performance in a format of 5 vs. 5 plus goalkeepers in IISs of 100 and 200 m^2^. On the other hand, Muñoz et al. [17] did not observe such differences in sprint performance over a distance of 15 m after SGs in similar formats. However, these authors [16] found lower sprint performance at a distance of 5 m after the SG. This loss of sprint performance could therefore be influenced by the SG format. Our results showed a loss of performance in the sprint at 10 and 30 m after the SGs were compared to the performance before the exercise, except for the 10 m sprints after SG2 and SG3. These results show that neuromuscular fatigue was appearing, manifested by the loss of performance in the ability to accelerate in SG1 (100 m^2^ and 4 * 6 min) and SG4 (200 m^2^ and 6 * 4 min) and to sprint in all formats of SG. The offside rule imposed in our investigation in order to ensure the SGs resembled the real game could have been an influence on the sprinting stabilization over the 10 m distance in SG2 and SG3. Commonly, the duration of breaks between the repetitions of training tasks is not taken into account in comparison to the results obtained in different investigations. However, the rest could influence the fatigue induced by SGs. In our study, the rest was two minutes between bouts, but Muñoz et al. [17] only included a rest of one minute between the bouts and they observed a loss of sprint performance over 15 m. Therefore, coaches should take into account the possibility that a rest of less than two minutes could be associated with higher fatigue.

In this study, no significant associations were found between the change in the sprint performances from pre-SG to post-SG and the internal load measured by the sRPEL recorded by players during the SGs. Taking into consideration these associations and the results reported by other authors [17], it seems that the PE is not a reliable indicator for assessing the neuromuscular fatigue caused during the playing of SGs. In our study, players declared the PE for whole bouts, and it is likely that this method does not reflect the neuromuscular fatigue induced by the SGs. Other researchers have observed that the fatigue suffered by soccer players during an SG could be caused by a neuromuscular component explained by the greater high-intensity and short-term actions in terms of changes of direction, acceleration, and deceleration occurring during the playing of the games [31]. Thus, in future research, it would be interesting to analyze the association between the external loads recorded during SGs and the loss of physical fitness in certain performance tests and indeed in other measurements of internal load (such as heart rate (HR) intensities) and biomarkers such as lactic acid, creatine kinase, or cytokines. Additionally, it would be interesting to analyze the supplementary effects on fatigue induced by different SG formats [32].

This study is not exempt from limitations, the main one being the sample of SGs, especially given the high variability that has previously been reported in soccer players’ running performances in matches [33]. Another limitation was the use of only one physical test to assess the neuromuscular fatigue induced by SGs; however, this information was jointly presented with the subjective effort declared by the young soccer players. A further limitation of our work is that it remains to be seen whether these findings can be extended to soccer players at the highest competitive levels. Finally, we were unable to quantify the effects of the SGs by applying other objective methods such as the measurement of HR and external load.

## 5. Conclusions

While no differences among the SGs in terms of internal load were found, a worse performance in the sprint test over 10 m and 30 m after every SG, except for the 10 m sprint in SG2 and SG3, was found. The findings revealed the appearance of neuromuscular fatigue, manifested by the loss of performance in the ability to accelerate in SG1 (i.e., 100 m^2^ and 4 * 6 min) and SG4 (i.e., 200 m^2^ and 6 * 4 min) and to sprint in all the SG formats. Therefore, these results could be useful for technical staff in constructing an effective design of the playing area and bout duration in their training tasks. Taking into account the aforementioned results, we suggest the use of these SG formats during acquisition sessions throughout the microcycle, especially in the central days of the week. In contrast, we advise against the use of this type of SG in the sessions before and after a match, because of the loss of the neuromuscular performance, which implies training-related fatigue in young soccer players.

## Figures and Tables

**Table 1:** Descriptive statistics and delta changes (Δ%, post-sided game value minus pre-sided game value as a percentage) in the players’ sprint performance measures, along with effect sizes (ES).

SG	Physical Measures	Pre-SG	Post-SG	Δ%	ES
SG1	Sprint 10 m	1.85 ± 0.07	1.95 ± 0.09 **	5.29	1.38
	Sprint 30 m	4.46 ± 0.13	4.74 ± 0.22 **	6.21	2.10
SG2	Sprint 10 m	1.90 ± 0.09	1.94 ± 0.13	1.94	0.43
	Sprint 30 m	4.58 ± 0.16	4.79 ± 0.23 **	4.54	1.32
SG3	Sprint 10 m	1.83 ± 0.11	1.89 ± 0.09	3.27	0.55
	Sprint 30 m	4.62 ± 0.22	4.77 ± 0.22 **	3.32	0.70
SG4	Sprint 10 m	1.87 ± 0.07	1.92 ± 0.08 **	2.73	0.74
	Sprint 30 m	4.58 ± 0.20	4.74 ± 0.21 **	3.40	0.78

SG: sided game; SG1: sided game played in an IIS of 100 m^2^ in 4 bouts of 6 min; SG2: sided game played in an IIS of 200 m^2^ in 4 bouts of 6 min; SG3: sided game played in an IIS of 100 m^2^ in 6 bouts of 4 min; and SG4: sided game played in an IIS of 200 m^2^ in 6 bouts of 4 min. ** Significant differences set at *p* < 0.01.
